# CDC20 regulates the cell proliferation and radiosensitivity of P53 mutant HCC cells through the Bcl-2/Bax pathway

**DOI:** 10.7150/ijbs.64003

**Published:** 2021-08-19

**Authors:** Shuai Zhao, Yichi Zhang, Xiuqin Lu, Han Ding, Bing Han, Xiaoling Song, Huijie Miao, Xuya Cui, Shiyin Wei, Wangrui Liu, Shuxian Chen, Jian Wang

**Affiliations:** 1 Department of Transplantation, Xinhua Hospital affiliated to Shanghai Jiao Tong University School of Medicine, Shanghai, China.; 2 Department of General Surgery, Xinhua Hospital affiliated to Shanghai Jiao Tong University School of Medicine, Shanghai, China.; 3 Shanghai University of Medicine & Health Sciences, Shanghai, P.R. China.; 4 Department of Oncology, Xinhua Hospital affiliated to Shanghai Jiao Tong University School of Medicine, Shanghai, China.; 5 Department of Neurosurgery, Affiliated Hospital of Youjiang Medical University for Nationalities, Guangxi, 533000, China.

**Keywords:** CDC20, P53 mutant, hepatocellular carcinoma, Bcl-2/Bax pathway

## Abstract

**Purpose:** The incidence of hepatocellular carcinoma (HCC) is extremely high, and China accounts for approximately 50% of global liver cancer cases. Previous studies reported that CDC20 is involved in the occurrence and progression of a variety of malignant tumors. So, whether CDC20 will affect the development of HCC, we have conducted in-depth research on this.

**Methods:** We selected Hep3B and HepG2 for cell culture, and performed siRNA transfection, lentiviral infection, western blot, MTS determination, cell cycle determination, apoptosis test, immunodeficiency test, clone survival test and subcutaneous parthenogenesis in nude mice.

**Results:** Knockdown of CDC20 greatly enhanced the radiation efficacy on the growth retardation in HepG2, and protein level of CDC20 was decreased for the activation of P53 by radiation. Downregulation of CDC20 combined with radiation can inhibit proliferation, aggravate DNA damage, increase G2/M arrest, and promote apoptosis of HCC cells to a greater extent, and the relative survival fraction of HCC cells was gradually reduced with radiation dose increased in P53 mutated Hep3B cells. After knocking down CDC20 in HCC, Bcl-2 was down-regulated and Bax expression increased. Down-regulation of CDC20 can inhibit further invasion by promoting the radiosensitivity of HCC.

**Conclusion:** In this study, we found that that CDC20 was highly expressed in HCC and participated in radio resistance of HCC cells with P53 mutation Bcl-2/Bax via signaling pathway. This study is the first to present evidence that CDC20 may play a role in improving the efficacy of radiotherapy in HCC.

## Introduction

Cancer is a serious public health issue with an enormous medical and financial burden for individuals and society. The incidence of HCC ranks among the highest in the world, with China accounting for about half of the total global cases; and HCC ranks fourth in the common cause of cancer-related deaths 1-3. There are many treatment methods for liver cancer, including surgical resection, radiotherapy, chemotherapy, gene therapy and immunotherapy, etc. Among these methods, surgical intervention is the most effective treatment; however, most HCC patients are diagnosed at middle to advanced stage and are therefore ineligible for surgery. Furthermore, HCC is highly resistant to chemotherapy and has a poor response to radiotherapy, which is partly ascribed to the high rate of genetic expression of drug resistance proteins and mutations in the p53 tumor suppressor gene 4-7. Therefore, better understanding the mechanisms of radio resistance in HCC is critical for improving the efficacy of radiotherapy and prolonging patient survival.

Currently, more than half of cancer patients need radiotherapy as an essential part of their cancer treatment 8-10. Studies have found that ionizing radiation (IR) can induce DNA double-strand breaks in target cells, thereby inhibiting cell proliferation and leading to cell apoptosis. The DNA damage response (DDR) is activated in response to IR, leading to repair of DNA damage and activation of the DNA damage checkpoint. Inhibition of DDR enhances the radiosensitivity of tumor cells, and DDR-based therapies targeting DDR-related molecules are a promising therapeutic strategy 11. The radio resistance of HCC to IR suggests that HCC cells have an unusual cellular repair ability closely related to the preferential activation of the DDR. The radiation sensitivity of tumor cells is affected by many factors, including proliferation, apoptosis, cycle distribution, and DDR 12, 13. Therefore, clarifying the factors and mechanisms that play a vital role is critical for improving the efficacy of radiotherapy in HCC.

Cell division cycle 20 homolog (CDC20) is a RING finger E3 ubiquitin ligase that functions as promoting complex/circular body (APC/C) activation in the later stage during the metaphase-anaphase transition and is one of the most important factors that control the spindle assembly checkpoint (SAC) 14. SAC ensures the correct separation of chromosomes during mitosis to maintain genome stability. In normally growing cells, when the SAC detects any defects in genomic stability, the cell cycle is arrested at metaphase. However, CDC20 overexpression allows cells with damaged DNA to leave mitosis and avoid apoptosis 15-17. Previous studies reported high levels of CDC20 in multiple malignancies and it participates in tumor generation and progression of tumor. Furthermore, genetic ablation of CDC20 caused elevated cellular apoptosis and blocked tumorigenesis *in vivo* 18-20.

In this study, we found that that CDC20 was highly expressed in HCC and participated in radio resistance of HCC cells with P53 mutation Bcl-2/Bax via signaling pathway. Our study first found the present evidence that CDC20 should be a significant role in improving the efficacy of radiotherapy in HCC.

## Materials and methods

### Cell culture

Two human HCC cell lines (Hep3B, HepG2) were purchased from the Chinese Academy of Medical Science. Cells were cultured in DMEM containing 10% fetal bovine serum (FBS) supplemented with 100 units/mL of penicillin and 100 μg/mL of streptomycin at 37 °C in a humidified atmosphere of 5% CO_2_. Collectting cells for experiments for the growth rate.

### siRNA transfection

CDC20 siRNA duplexes were transfected using Lipofectamine 3000 reagent (Invitrogen, USA). Cells were used for further analyses after transfection for 48 h. The sequences of the CDC20 siRNA duplexes are as follows: si1#: AACGGCAGGACUCCGGGCCGA, si2#: AAUGGCCAGUGGUGGUAAUGA.

### Lentiviral infection

CDC20 short hairpin RNA (shRNA)-expressing lentivirus and vector were purchased from Genecopoeia. The short hairpin RNA (shRNA) sequences targeting CDC20 are as follows: sh1#: AACGGCAGGACUCCGGGCCGA, sh2#: AAUGGCCAGUGGUGGUAAUGA. After 24 h infection with lentivirus, the cells were passaged and selected by 10 μg/mL puromycin (Sigma-Aldrich) for 48 h to eliminate the uninfected cells before harvesting for further analyses. Knockdown efficiency was determined by western blot.

### Western blot

Cells were harvested after the indicated treatments and dissolve RIPA buffer; protein concentrations quantification by bicinchoninic acid detection kit (Pierce, USA). Protein samples were Sodium dodecyl sulfate polyacrylamide gel electrophoresis (SDS-PAGE) separation under denaturing conditions and transferred to a nitrocellulose filter (NC) membrane. The membrane was then incubated with the following primary antibodies: anti-β-tubulin (CST), anti-GAPDH (CST), anti-CDC20 (Proteintech), anti-BCL-2 (CST), anti-BAX (CST), anti-p21 (Proteintech), anti-P53 (Proteintech), anti-CCNB1 (CST), anti-CCND1 (CST).

### MTS assay

MTS assay (Sigma-Aldrich) was performed to measure cell proliferation. Cells in each well were incubated with 20 μl of MTS solution (5 mg/mL) for 1 h at 37 °C. The optical density (OD) at the wavelength of 490 nm was measured by a multidetection microplate reader (BMG LABTECH, Cary, NC, USA).

### Cell cycle assay

The cells were washed 3 times with PBS, fixed with 70% ethanol at -20 °C for more than 12 hours, and stained with RNase (50 µg/mL) and PI for 30 minutes at room temperature. Then analyze the stained cells by flow cytometry (FC500 MPL, Beckman Coulter).

### EdU assay

EdU (5-ethynyl-20-deoxyuridine, Invitrogen) was used at 1 mM for 2 h in cell culture medium as described by the manufacturer (Invitrogen). Hoechst® 33342 solution was used to stain the nuclei. Cells were mounted and imaged using fluorescence microscopy.

### Immunofluorescence staining

Cells were fixed with 4% paraformaldehyde solution for 15 minutes, permeabilized with 0.5% Triton X-100 for 15 minutes, blocked with 1% BSA for 30 minutes, and then with Anti-γ H2AX (S139) (Millipore, 1:500 diluted in 1% In BSA) for 1 hour at 37 °C. After washing 3 times with PBS, the cells were incubated with the secondary antibody for 1 hour at 37 °C and stained with DAPI to reveal the nucleus. Capture and visualize fluorescence images under a high-content imaging system (Operetta, Perkin Elmer).

### Clonogenic survival assay

Cells were infected with the indicated lentivirus and screened by puromycin at 2 μg/ml at 48 h after infection for at least one week. Cells were then exposed to the indicated doses of IR, and 1000 cells from each condition were re-plated and incubated at 37 °C with 5% CO_2_ for 10 days. The colonies were fixed with 4% paraformaldehyde and stained with 0.1% crystal violet. Colonies consisting of >50 cells were counted. The surviving fraction was defined as the plating efficiency at the experimental irradiation dose divided by that at 0 Gy. The cell survival curve was plotted by single-hit multitarget modeling and linear quadratic simulation.

### Subcutaneous nude mouse model

Twelve BALB/c nude mice were randomly separated into two groups: the shNC group and the shCDC20 group (n=6/group). Hep3B cells (1 × 10^6^ in 200 μL) infected with either shNC or shCDC20 lentivirus were subcutaneously injected into the right flank of the mice. The size and volume of tumors were measured every 3 days. When the tumors reached approximately 50 mm^3^, three mice in each group were exposed to 4 Gy and the other three mice were allocated to the control group (0 Gy). The tumor sizes were measured every 7 days and the tumor volumes were calculated: V (cm^3^) = width^2^ (cm^2^) * length (cm) / 2. Mice were euthanized on day 35.

### Statistical analysis

Statistical analyses were performed using GraphPad Prism 8.0 (GraphPad Software, CA, USA) and SPSS 20.0 (IBM Corp., NY, USA). Data are expressed as mean ± standard deviation (SD). Student's t-test was used to identify differences between pairs of group means. One-way ANOVA with Bonferroni's test was used to compare more than two group means. p < 0.05 indicated statistical significance.

## Results

### CDC20 is highly expressed and serves as an unfavorable prognosis marker in HCC

**Figure 1:**
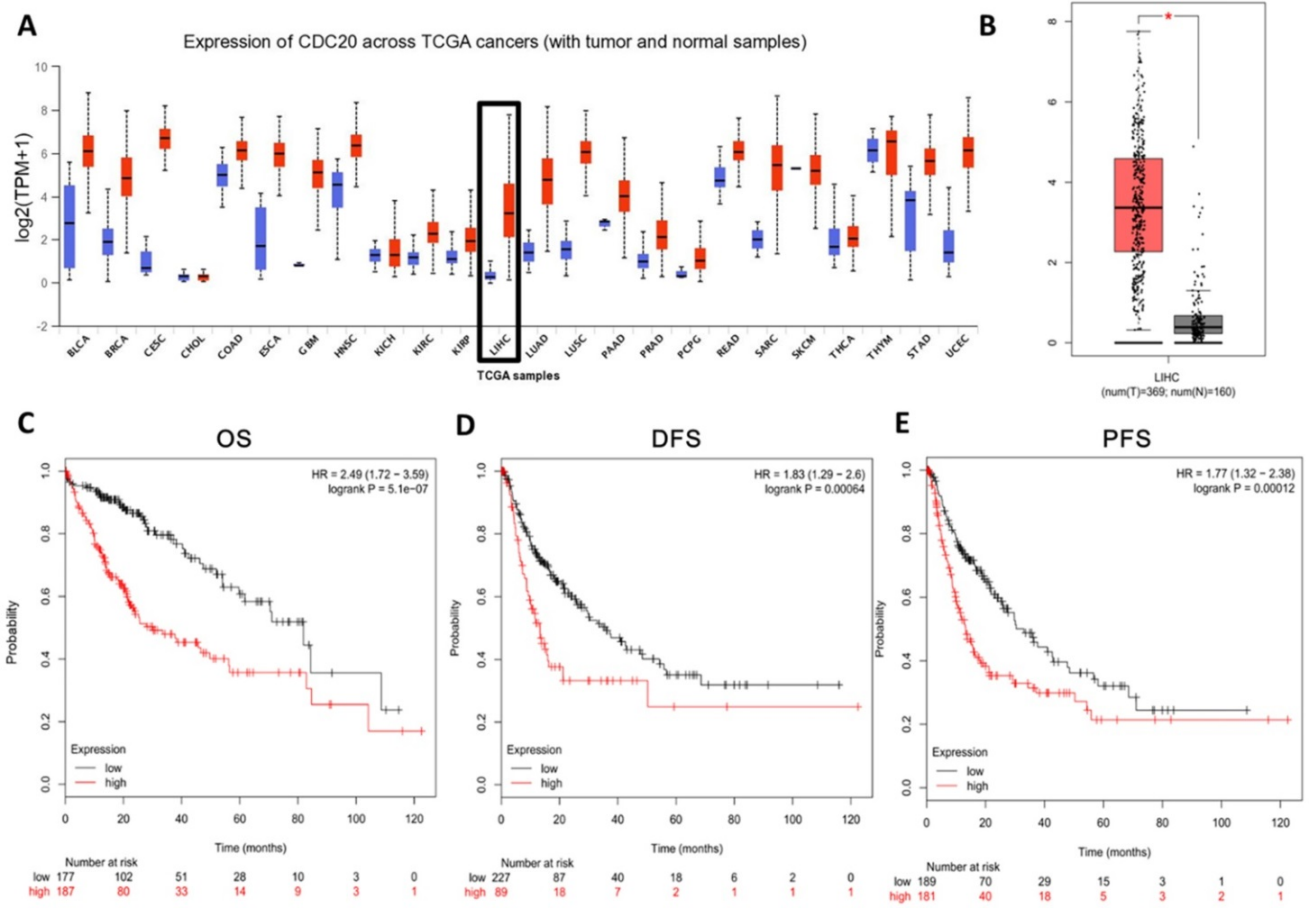
** CDC20 is highly upregulated and served as an unfavourable prognosis marker in HCC samples. A.** Expression of CDC20 mRNA among different tumor tissues (red, tumor samples; blue, normal samples). **B.** Expression of CDC20 in liver cancer. **C-E.** Correlation analysis of the expression of CDC20 between clinic OS. (C) DFS, (D) PFS and (E) in HCC samples. *P < 0.05 indicated difference had statistical significance.

In order to study the role of CDC20 in HCC, we analyzed the CDC20 mRNA levels in tumor tissues and non-tumor tissues of HCC patients. CDC20 was upregulated in many types of cancers (**Figure 1A**) including HCC (**Figure 1B**) according to the GEO database through UALCAN and GEPIA. Next, we analyzed the relationship between the expression of CDC20 and the prognosis of HCC patients, and the results indicated that high CDC20 expression was correlated with poor overall survival (OS) (**Figure 1C**), disease-free survival (DFS) (**Figure 1D**), and progression-free survival (PFS) in HCC (**Figure 1E**). Collectively, these results indicated that CDC20 is highly expressed in HCC, and its high expression is associated with poor prognosis in HCC.

### Bioinformatics analysis of CDC20 and its related genes in HCC

**Figure 2:**
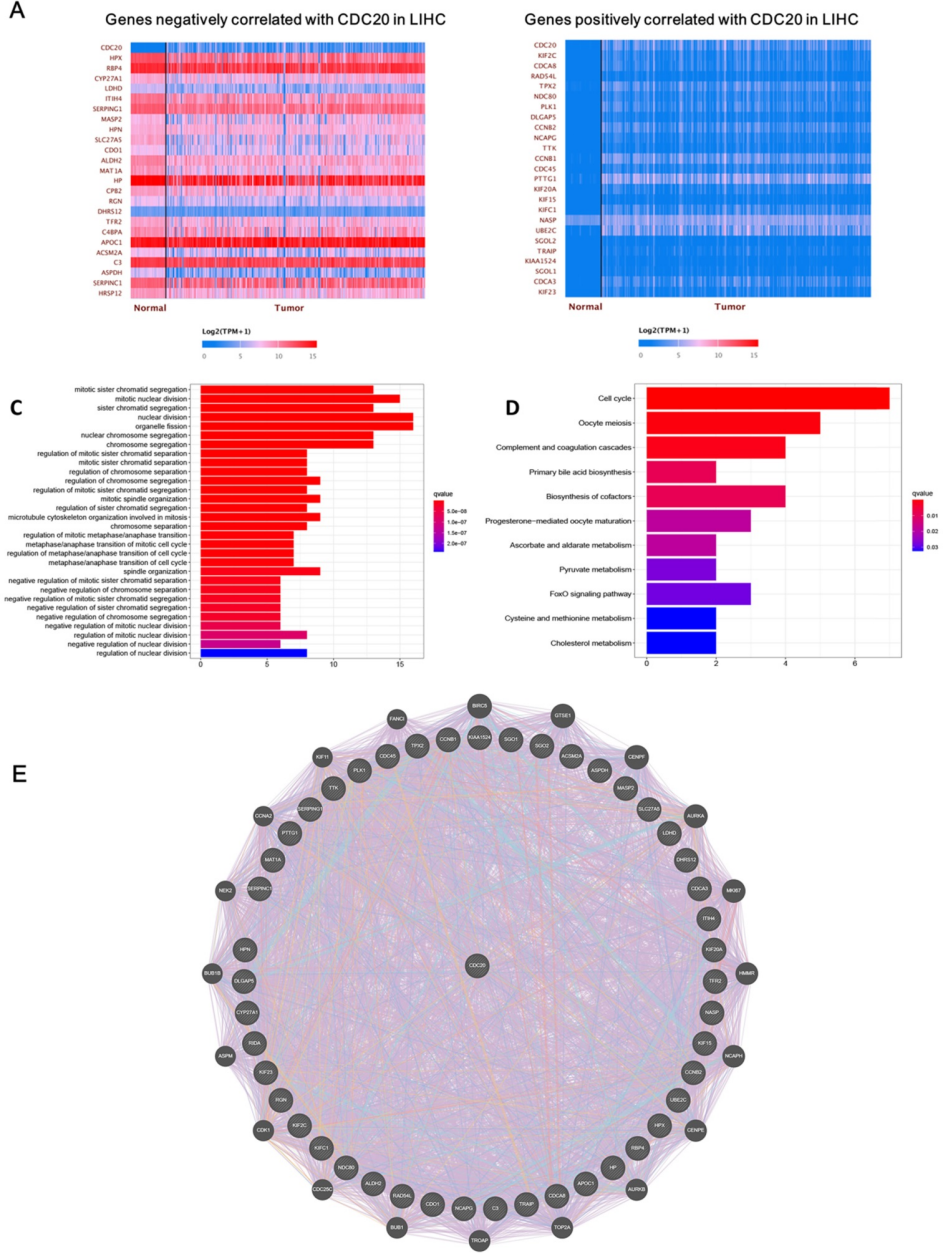
** CDC20 related DNA damage pathway in HCC. A-B.** The top 25 significant genes set positively and negatively correlated with CDC20 as shown in the heat map. **C-E.** KEGG pathway analysis showed enrichment biological process of the DNA replication, DNA damage, spindle assembly, and cell cycle regulation.

Next, we analyzed the biological functions of genes related to CDC20. After screening, we found that 4,622 genes were positively correlated with CDC20, while 57 genes were negatively correlated with CDC20. The top 25 significant genes that were positively or negatively correlated with CDC20 are shown in the **Figure 2A-B**. GO term analysis showed that the significant genes correlated with CDC20 were involved in mitotic sister chromatid segregation, mitotic nuclear division, and sister chromatid segregation (**Figure 2C**). KEGG pathway analysis showed genes were enriched in DNA replication, DNA damage and cell cycle pathways (**Figure 2D**). Gene-gene interactions between clinical-related DEGs and related genes were analyzed, as illustrated in **Figure 2E**. Approximately 84.18% of terms were in co-expression and 6.87% terms were in co-localization.

### CDC20 suppression suppresses cell proliferation, upregulates apoptosis, and induces G2/M phase cell cycle arrest of HCC cells

**Figure 3:**
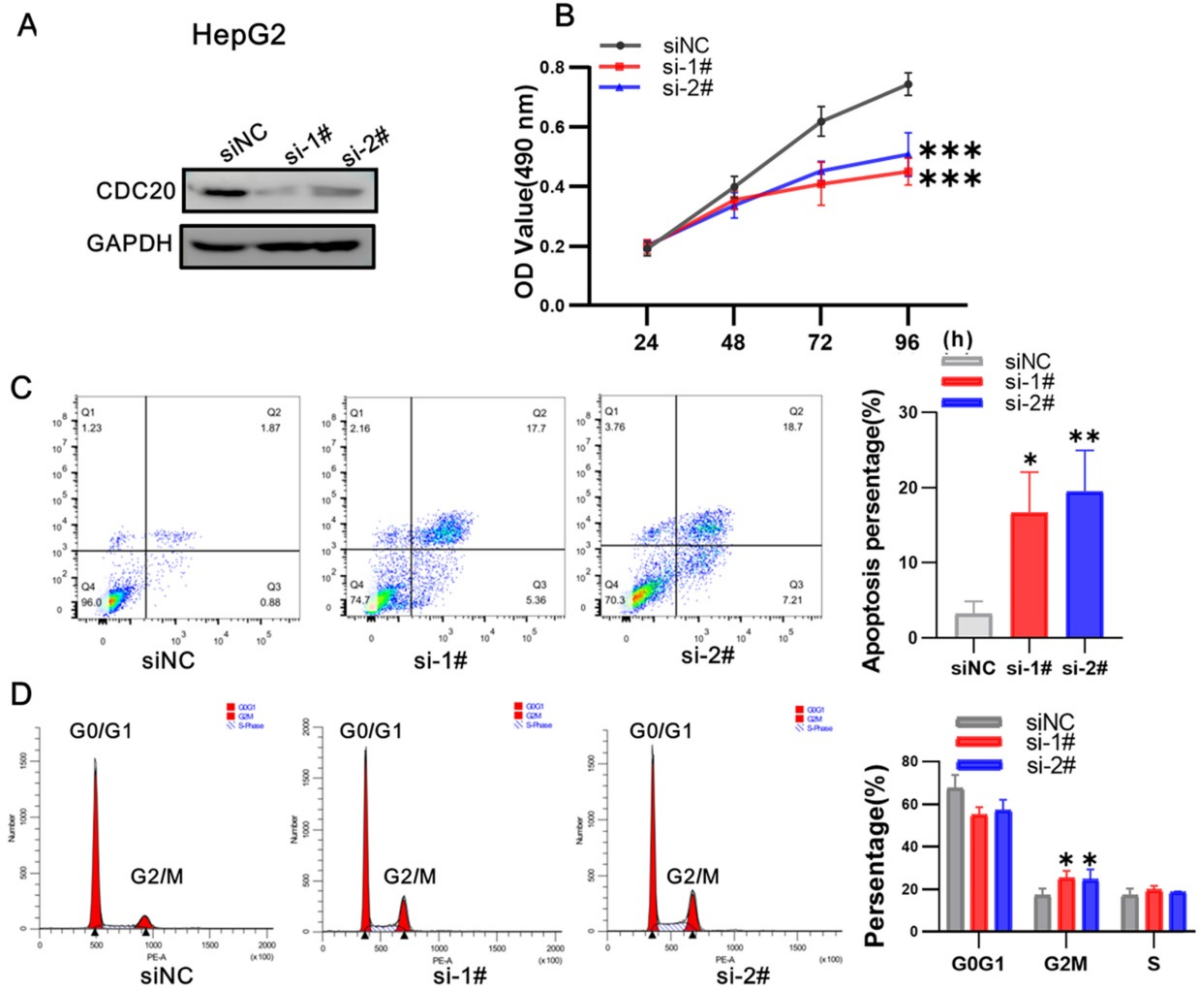
Downregulation of CDC20 suppressed proliferation, promoted apoptosis, and exacerbated cell cycle arrest at the G2/M phase of HepG2 cells. **A.** The protein level of CDC20, apoptosis and cell cycle related proteins with different treatments was detected by WB. **B.** MTS assays detected the cell proliferation of each group. **C.** The apoptosis percentages of each group were measured by flow cytometry. **D.** Flow cytometry was used to determine the percentage of cell cycle. Data are presented as means ± SD. *P < 0.01 indicated difference had statistical significance versus the siNC.

We next used siRNA targeting CDC20 to knock down the expression of CDC20 in two HCC cell lines, HepG2 and Hep3B. The research on Hep G2 was first conducted. Western blot showed that CDC20 protein expression was markedly decreased after siRNA-CDC20 transfection, compared with the negative control group (siNC) (**Figure 3A**). We next conducted MTS assays to investigate the effect of CDC20 knockdown on HCC cell proliferation. Proliferation was significantly inhibited in both HepG2 and Hep3B siRNA-CDC20 cells compared with controls (p <0.01) (**Figure 3B**). Compared with the siNC group, both siRNA- CDC20 cell groups showed increased apoptosis and G2/M phase arrest (p <0.01), and 30% cells in the G2/M phase (**Figure 3CD**).

**Figure 4:**
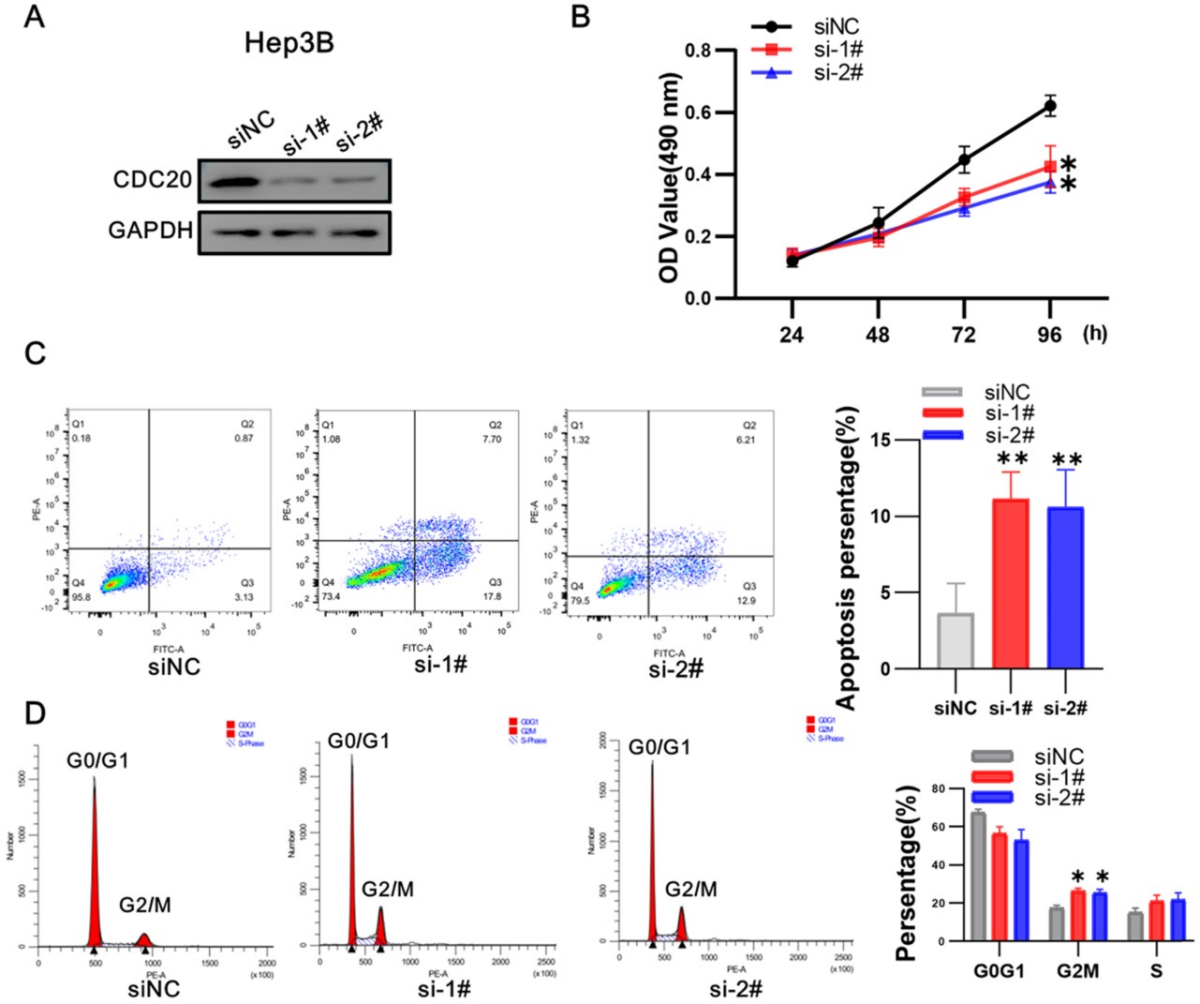
Downregulation of CDC20 also significantly suppressed proliferation, promoted apoptosis, and increased cell cycle arrest at the G2/M phase of Hep3B cells. **A.** The protein level of CDC20, apoptosis and cell cycle related proteins with different treatments was detected by WB. **B.** MTS assays detected the cell proliferation of each group. **C.** The apoptosis percentages of each group were measured by flow cytometry. **D.** Flow cytometry was used to determine the percentage of cell cycle. Data are presented as means ± SD. *P < 0.01 indicated difference had statistical significance versus the siNC.

After that, we conducted research on Hep 3B. Western blot showed that CDC20 protein expression was markedly decreased after siRNA-CDC20 transfection, compared with the negative control group (siNC) (**Figure 4A**). We next conducted MTS assays to investigate the effect of CDC20 knockdown on HCC cell proliferation. Proliferation was significantly inhibited in both HepG2 and Hep3B siRNA-CDC20 cells compared with controls (p <0.01) (**Figure 4B**). We used flow cytometry to evaluate the effect of CDC20 inhibition on the apoptosis and cell cycle of HCC tumor cells. Compared with the siNC group, both siRNA- CDC20 cell groups showed increased apoptosis and G2/M phase arrest (p <0.01), and 30% cells in the G2/M phase (**Figure 4C-D**). The results suggested that silencing CDC20 decreases cell proliferation, increases apoptosis, and induces the G2/M phase cell cycle arrest of HCC cells.

Western blot assays revealed that the levels of pro-apoptosis protein Bax and G2/M phase-related protein cyclin B1 (CCNB1) were significantly higher in the siCDC20 groups. The siCDC20 groups also showed significant reduction of anti-apoptotic Bcl-2 protein and G2/M phase-related protein cyclin D1 (CCND1) compared with the siNC group. These results suggested that the influence of CDC20 inhibition of cell proliferation, apoptosis, and G2/M phase cell cycle arrest of HCC cells may be through the cell cycle- and apoptosis-related pathway.

### CDC20 suppression significantly suppressed cell proliferation of Hep3B cells but only moderately suppressed HepG2 cell proliferation when combined with x-ray irradiation

**Figure 5:**
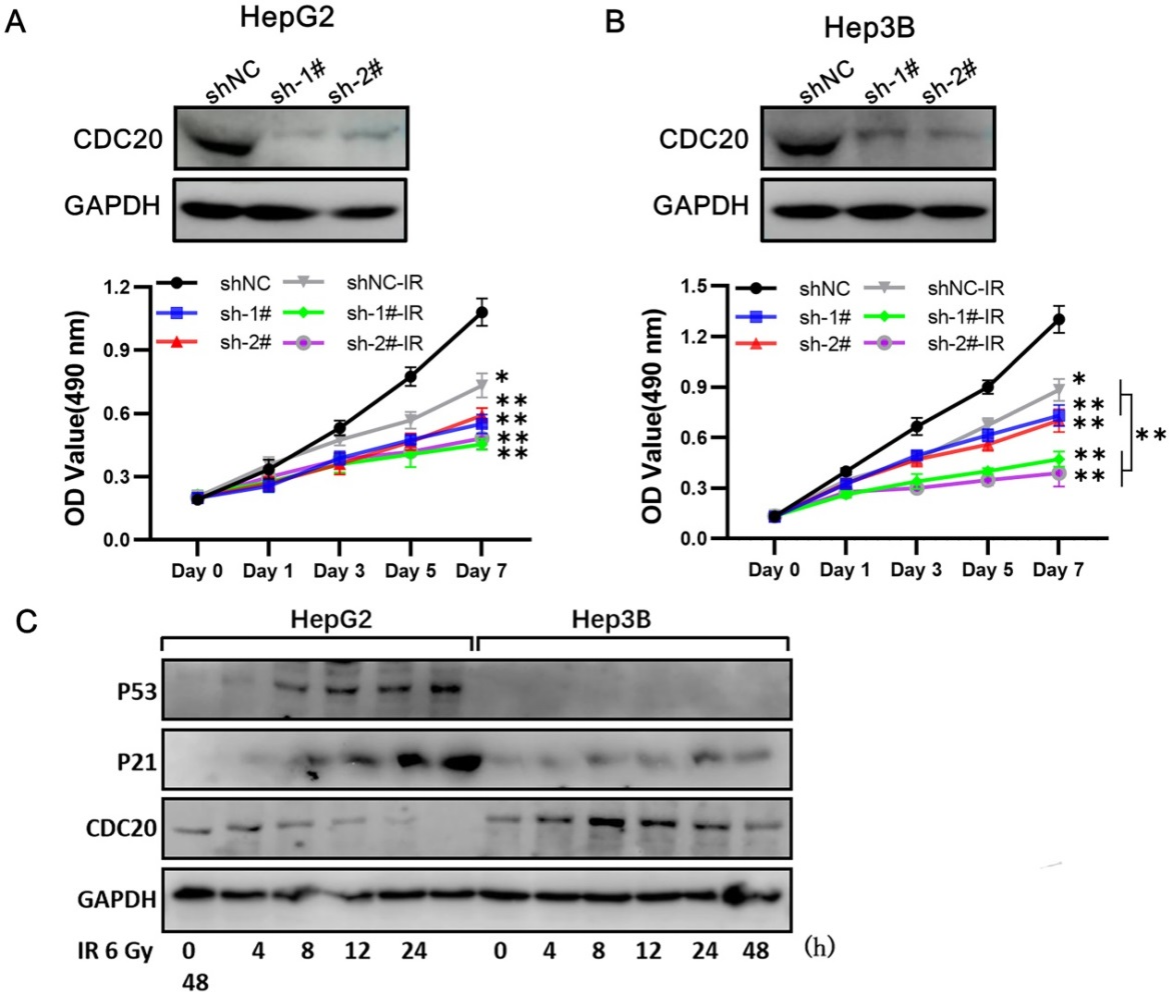
When combined with x-ray irradiation, CDC20 suppression significantly suppressed cell proliferation of the cell of Hep3B, but only moderately of HepG2. **A&B.** The protein expression level of CDC20 detected by WB. Relative proliferation ability of the two HCC cell lines after different doses of IR was detected by MTs assay. **C&D.** The protein level of P53, P21 and CDC20 were detected by WB at various time points. P53-WT HepG2 cells and Hep3B cells treated with 6 Gy IR. Data are presented as means ± SD. *P < 0.05, **P < 0.01 versus the shNC.

At 3 days after infection with shRNA-CDC20 or shNC lentivirus, Hep3B and HepG2 cell lines were irradiated with 6 Gy. We found that CDC20 suppression significantly suppressed cell proliferation of Hep3B cells; the cells were almost stop growing since day 3 when radiotherapy combined with CDC20 downregulation (**Figure 5A**, p < 0.01). However, the same phenomenon was not exhibited in HepG2 cells. Radiotherapy combined with CDC20 downregulation resulted in only a moderate growth inhibition of HepG2 cells compared with cells receiving radiotherapy with shNC (**Figure 5B**).

A previous study reported that CDC20 is negatively regulated by p53 in lung cancer cell lines 15. We found that p53 and p21 expressions were increased at 8 h after treatment with 6 Gy in the wild-type p53 HepG2 cells and CDC20 expression was gradually reduced (**Figure 5C**). These results indicated that CDC20 suppression significantly suppressed cell proliferation of p53 mutant Hep3B cells, but only moderately suppressed wild-type p53 HepG2 cell proliferation when combined with irradiation.

### CDC20 suppression enhances the sensitivity of Hep3B cells to radiation

**Figure 6:**
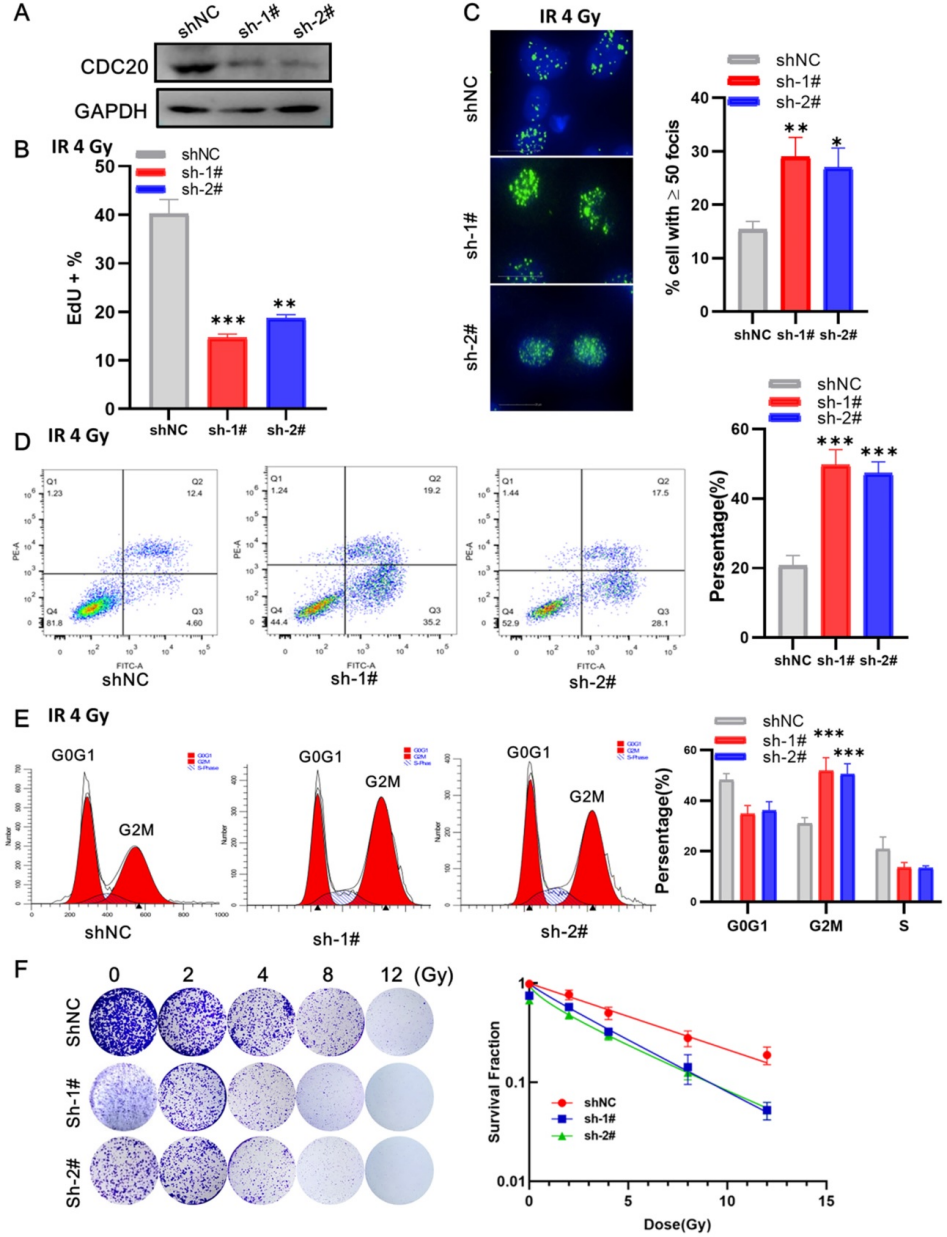
CDC20 knockdown promotes radiosensitivity of Hep3B cells. **A.** The protein expression level of CDC20 detected by WB. **B.** The cell proliferation of each group detected by EdU assay. **C.** The number of γH2AX foci measured by High-Content Imaging System. **D.** Apoptotic cells analyzed by flow cytometry 24 h after irradiation. **E.** Flow cytometry was used to determine the percentage of cells in G1, S and G2/M phase. **F.** Representative crystal violet staining of the colonies formed by Hep3B cells treated with control, and shCDC20 combined with irradiation. Data are presented as means ± SD. *P < 0.05, **P < 0.01, ***P < 0.001 versus the shNC.

Next, we examined the impact of CDC20 suppression combined with radiation in p53 wild-type Hep3B cells. At 3 days after lentivirus infection, the cells were irradiated with 6 Gy irradiation. EdU assay showed that cells with CDC20 knockdown had a lower proliferation rate than control cells (**Figure 6A-B**, p < 0.01). The number of γH2AX lesions in shCDC20 cells at 24 hours after IR was significantly higher than that in control cells (**Figure 6C**, p < 0.01). Strikingly, the combination of IR and CDC20 suppression induced further apoptosis (**Figure 6D**, p < 0.01) and G2/M phase arrest compared with G0G1 (**Figure 6E**, p < 0.01). The radiosensitizing effects of CDC20 knockdown were measured in cells subjected to 0, 2, 4, 8, and 12 Gy using colony-forming assays (**Figure 6F**). The colony formation ability of cells exposed to ≥ 4 Gy was significantly different from the non-irradiated cells. The survival curve of the IR + shCDC20 group was significantly shifted downward compared with non-irradiated cells (**Figure 6F**, p < 0.01).

### Downregulation of CDC20 results in upregulated Bax and decreased Bcl-2 in HCC cells after radiotherapy

**Figure 7:**
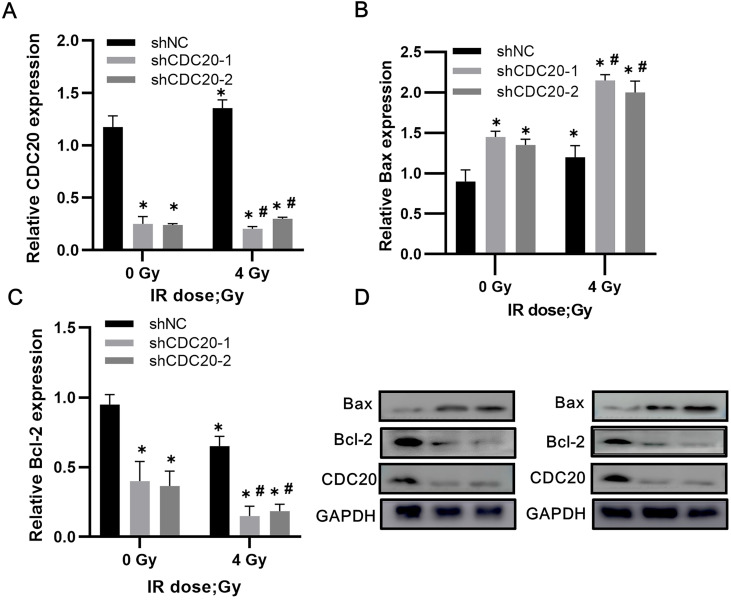
Reduced CDC20 upregulates Bax and declines Bcl-2 in HCC cells after radiotherapy. **A-C.** Protein expression of CDC20, Bax and Bcl-2 expression of Hep3B cells in each group before and after radiotherapy. **D.** Protein electrophoregrams of each group. * P < 0.05 vs the shNC group at dose of 0 Gy, ^#^ P < 0.05 vs at dose of 4 Gy in the same infected cells.

Western blot analysis of non-transfected Hep3B cells showed increased CDC20 expression after 4 Gy of irradiation, with slightly reduced expression of Bax and increased Bcl-2 expression. In the CDC20 knockdown group, Bax increased (p < 0.05) and Bcl-2 decreased (p < 0.05) after 4 Gy compared with the shNC group with or without IR (**Figure 7A-D**).

### Down-regulated CDC20 represses tumorigenesis by promoting HCC radiosensitivity *in vivo*

To validate the *in vitro* results, the effect of CDC20 on radiosensitivity was investigated in a mouse model of HCC injected with Hep3B cells. The tumor growth curve is shown in **Figure 7**. The tumor growth and tumor volumes of the shNC group and the shCDC20 group were significantly suppressed after 4 Gy IR compared with mice in the respective control groups treated with 0 Gy. Furthermore, the tumor volume of the shCDC20 group was decreased compared with the shNC group (P < 0.05).

### CDC20 suppresses tumor proliferation and increases radiotherapy sensitivity in the HCC xenograft mouse model

**Figure 8:**
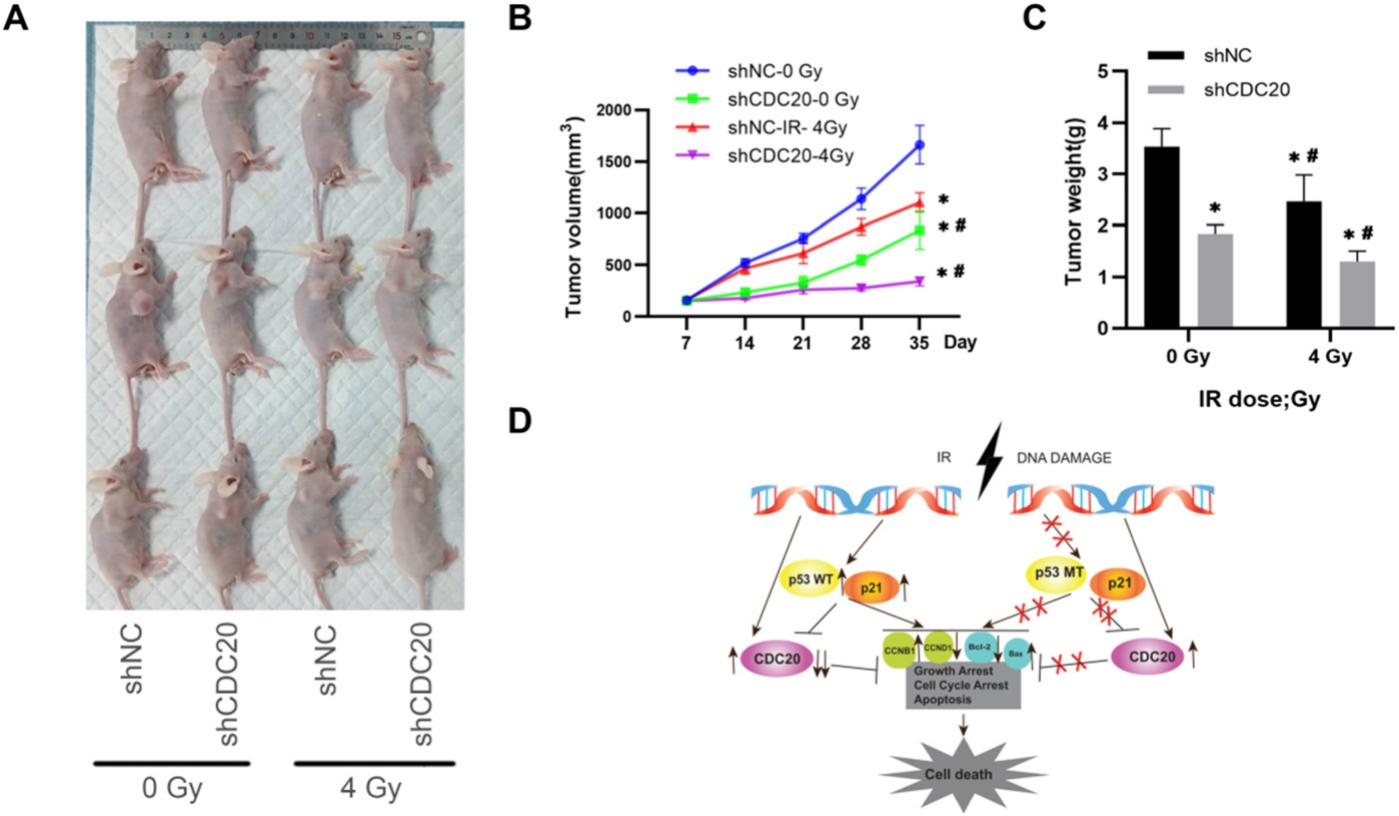
Down-regulated CDC20 represses tumorigenesis by promoting HCC radiosensitivity *in vivo*. **A.** Tumor growth curve of mice in each group with or without insfection and radiotherapy. **B.** Tumor volumes of mice in each group. **C.** The images of tumors of mice in each group; * P < 0.05 vs the shNC group, # P < 0.05 vs at dose of 0 Gy in the same transfected cells (12 mice, 4 groups, shnc, shcdc20,IR+ shnc, IR+ shcdc20).

In order to further verify the role of CDC20 in the proliferation of HCC cells, we injected nude mice under the skin to construct a xenotransplanted mouse model. When tumors reached 120 mm^3^, mice were randomized into two groups (n = 6 each) and treated with 4 Gy radiotherapy (**Figure 8A**). Tumor size and volume were significantly inhibited in the shCDC20 group compared with the control (**Figure 8B-C**). Additionally, in suggested that CDC20 could significantly enhance sensitivity of HCC cells receiving radiotherapy (p<0.05).

These results indicated that CDC20 suppression significantly suppressed cell proliferation of p53 mutant Hep3B cells, but only moderately suppressed proliferation of p53 wild-type HepG2 cells when combined with x-ray irradiation. This mechanism may be related to the induction of wild-type p53 and increase of p21 caused by IR, leading to suppression of CDC20 expression and apoptosis and G2/M arrest by BCL/BAX and the CCNB1/CCND1 pathway in p53 wild-type HepG2 cells. Mutation in p53 impairs its regulation of CDC20, as well as growth arrest and apoptosis. The protein level of CDC20 was increased in p53 mutant Hep3B cell lines after IR (**Figure 8D**).

### Immunotherapy efficacy and TIME characterizations analysis of RBCK1

**Figure 9:**
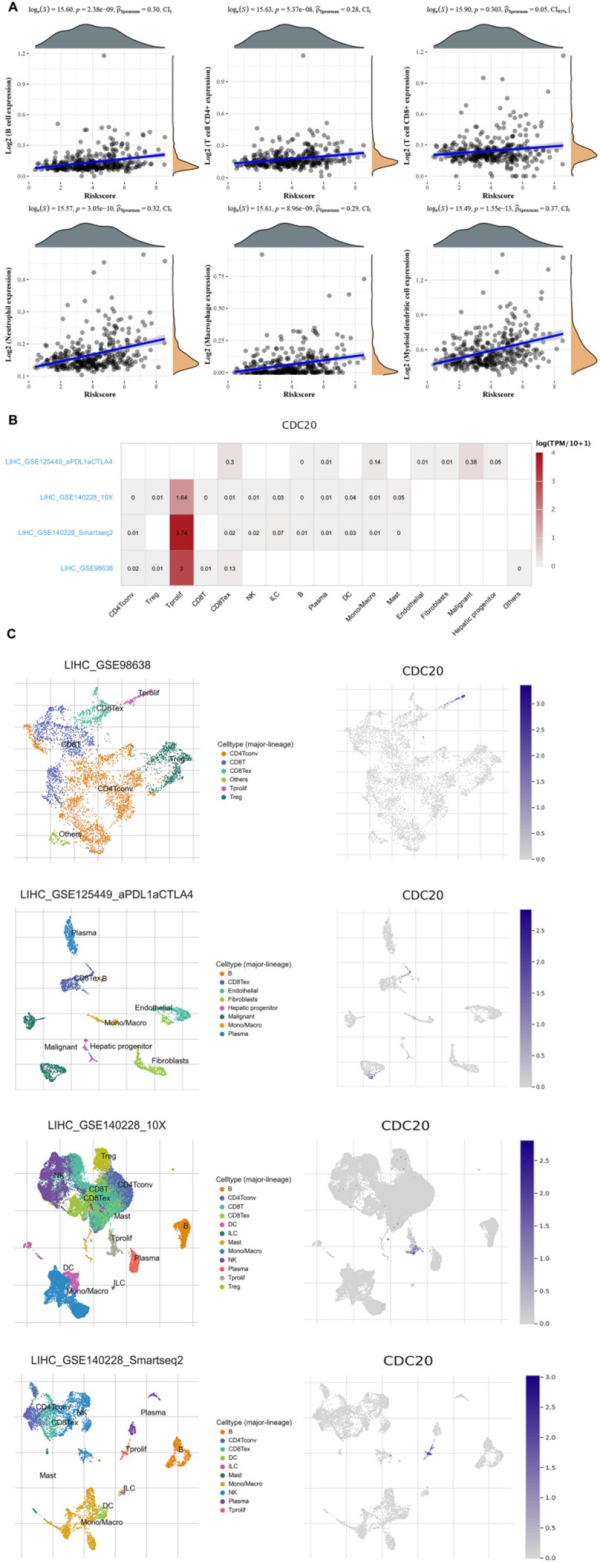
Correlation analysis of CDC20 in the immune microenvironment of HCC. **A.** Spearman correlation analysis of CDC20 expression and score. The horizontal axis in the figure represents the expression distribution of the gene, and the ordinate is the expression distribution of the score. The density curve on the right represents the distribution trend of the score;the upper density curve represents the distribution trend of the gene;the top side The value represents the correlation p value, correlation coefficient and correlation calculation method. **B.** We quantitatively calculated the positioning and binding of CD3E on various immune cells across the dataset using a heatmap. **C.** Four single-cell RNA-seq datasets were enrolled to determine the location of CD3E in different cell.

Finally, we analyzed the role of CDC20 in the immune microenvironment of HCC. We verified the Spearman correlation analysis between CDC20 and immune score. We found that in HCC, the expression of CDC20 is closely related to B cell expression, T cell CD4+ expression, T cell CD8+ expression, Neutrophil expression, Macrophage expression, Myeloid dendritic cell expression (p<0.001) (**Figure 9A**). Then, we analyzed the expression distribution of CDC20 in HCC cell lines. We found that CDC20 is highly expressed in HLF, SNU-387, SNU-423 and other cell lines (**Figure 9B**). A heatmap showed the relatively high expression of a CDC20 in different cell types across three scRNA-seq datasets (**Figure 9C**). In order to reduce the heterogeneity of tumor tissues, and to further confirm localization of CDC20 in immune cells, we included and analyzed three single-cell RNA-seq (scRNA-seq) datasets, GSE98638, GSE125449, GSE140228_10X and GSE140228. As shown in **Figure 9D**, CDC20 is mainly located or bind to Tprolif.

### CDC20 significantly predicts survival and aggressive clinicopathological parameters for HCC patients

**Figure 10:**
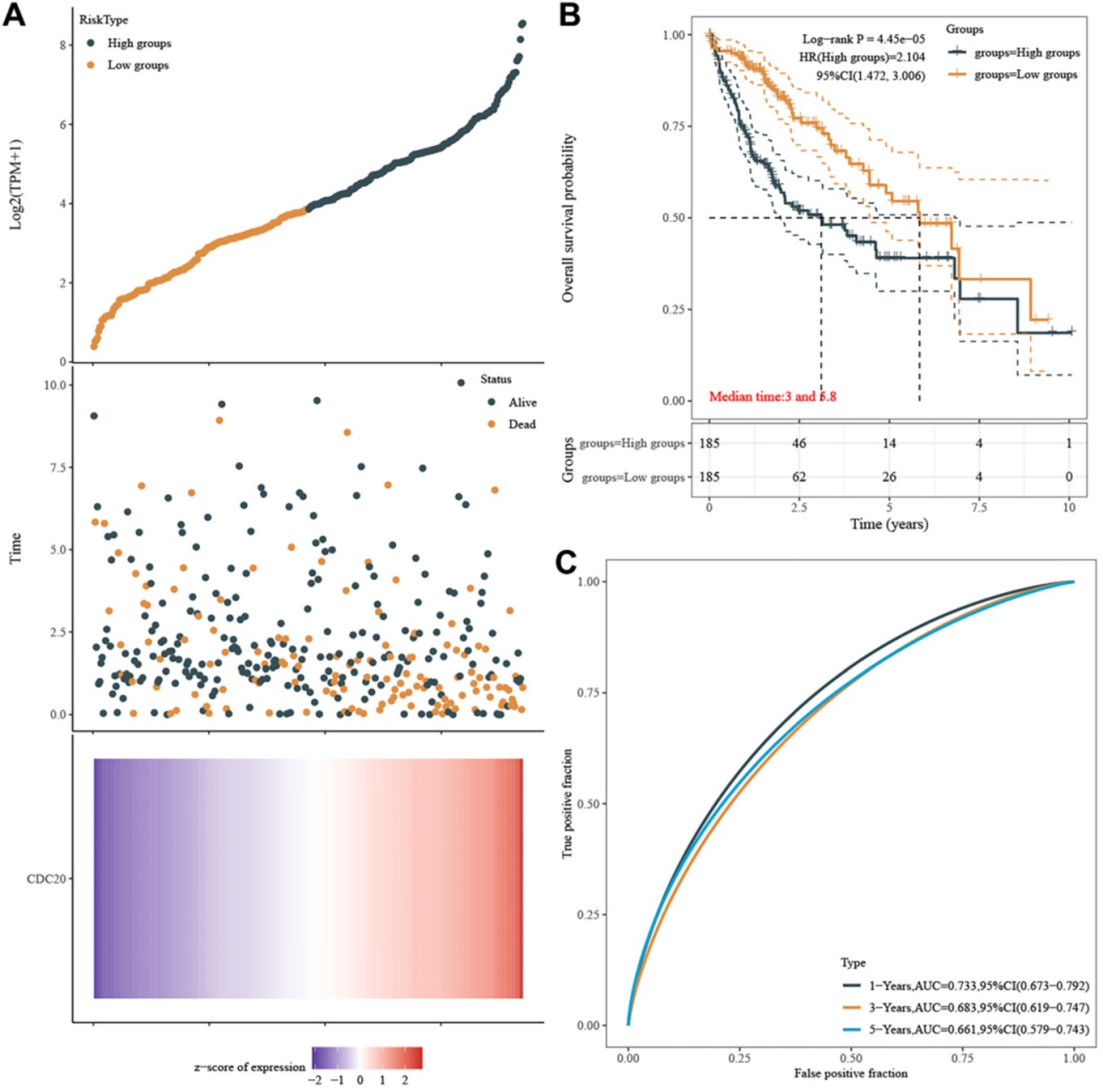
CDC20 significantly predicts survival. **A.** HCC patients with higher CDC20 expression significantly experience higher risk of death and the z-score of expression confirmed that high expression of CDC20 was associated with higher mortality. **B.** Kaplan-Meier curve proved that high CDC20 expression was significantly correlated with worse OS, with median survival equal 2.8 years in CDC20^high^ group and 6.3 years in CDC20^low^ group. **C.** ROC curve proved high sensitivity and specificity of the independently diagnostic and prognostic value of CDC20 expression.

Finally, we explored the relationship between the expression of CDC20 and the survival of HCC patients. HCC patients with higher CDC20 expression experienced a significantly increased risk of death, and the z-score of CDC20 expression confirmed that elevated CDC20 levels were associated with higher mortality (**Figure 10A**). The Kaplan-Meier curve also demonstrated that high CDC20 expression led to worse OS in 371 patients, with a median survival of 2.8 years in the CDC20^high^ group and 6.3 years in the CDC20^low^ group (**Figure 10B**). The high sensitivity and specificity of the independent diagnostic and prognostic value of CDC20 expression were shown by the ROC curve (**Figure 10C**). However, the accuracy decreased over time [1-year area under the curve (AUC)=0.733, 3-year AUC=0.683 and 5-year AUC=0.661, suggesting that CDC20 expression more accurately predicted the prognosis of early-stage HCC patients compared with those with advanced stages.

## Discussion

In our research, we have found that CDC20 is closely related to the prognosis of HCC, and inhibiting CDC20 can enhance the radiosensitivity of HCC cells, and thus have new ideas in the choice of treatment of HCC patients. Biomarkers for predicting the prognosis of HCC patients open up new directions. As a regulator of the spindle checkpoint, CDC20 plays an important role in regulating the activity of the all-ubiquitin ligase of APC/C. CDC20 has been found to be a potential therapeutic target and biomarker for a variety of cancers. Studies have found that after CDC20 is inhibited, the growth of cancer cells will be inhibited, G2/M phase arrest and apoptosis 21. In our research, we also found that the high expression of CDC20 will lead to a worse prognosis of HCC patients; while inhibiting CDC20 will increase the radiosensitivity of HCC tumor cells, thus providing a new direction and idea for us to treat HCC 22, 23.

Studies have shown that mutations in CDC20 can prevent cell division during mitosis, resulting in failure to start later, making chromosomes impossible to separate. Studies have found that CDC20 also plays a key role in mediating protein-protein interactions. These may be related to the influence of HCC's radiosensitivity. Interestingly, it is also suggested that Cdc20 has a function independent of APC/C. Most of the previous studies mainly focused on the analysis of the function of CDC20 and its downstream ubiquitin targets, while the analysis of upstream regulators of CDC20 and related up-regulated genes is rare. In our research, we found that CDC20, its related upstream regulators and related genes mainly play a role in mitotic sister chromatid segregation, mitotic nuclear division, sister chromatid segregation, DNA replication, DNA damage and cell cycle pathways. In addition, the research team also observed that p53 inhibits tumor cell growth by regulating Cdc20. Therefore, we further study the relationship between P53 and CDC20 in HCC.

The rapid development of modern bioinformatics and phenotyping has provided great convenience to our research 24-26. Tumor formation is a complex and multistep process that involves multiple factors and gene alterations over various stages. Two important hallmarks in the development of tumor cells are abnormal cell cycle regulation and dysregulated cell proliferation 27, 28. Proper cell cycle regulation is critical for cell survival, proliferation, and growth, and cell cycle proteins are targets of anticancer therapy. Cell cycle arrest is also closely associated with the occurrence of tumor and cellular senescence 29-31. Flow cytometry revealed that CDC20 suppression increased cell cycle arrest at the G2/M phase in HCC cells, and HCC cell proliferation was significantly decreased after inhibition of CDC20 expression by siRNA-CDC20 transfection. Apoptosis or programmed cell death is an integral part of cell maintenance^32^ and plays an important role in tumor cell growth, immunity, metabolism and clearance. The G2/M cell cycle arrest prevents tumor cells from entering mitosis, which hinders tumor progression. After inhibition of CDC20, HCC cells showed a G2/M arrest, thus inhibiting tumor cells from entering mitosis and leading to osteosarcoma deterioration. Western blot analysis of tumor tissues showed that irradiation combined with CDC20 inhibition resulted in upregulated pro-apoptosis protein Bax and downregulated anti-apoptosis protein Bcl-2 33-35. We speculate that wild-type p53 activated by IR induces p21 and suppresses the expression of CDC20, leading to apoptosis and G2/M arrest. In contrast, mutant p53 shows impaired ability to regulate CDC20, as well as growth arrest and apoptosis.

Radiation therapy is the one of the most common cancer treatments. The most important finding in this study is that the CDC20 suppression improved the radiosensitivity of HCC cells. However, our results showed that CDC20 suppression combined with x-ray irradiation significantly suppressed cell proliferation of Hep3B cells, but only moderately suppressed HepG2 cell proliferation. CDC20 expression is suppressed by genotoxic stress in p53- and p21-dependent manners. We found accumulation of p53 and p21 proteins and suppression of CDC20 in p53-wildtype HepG2 cell lines treated with IR. In contrast, the protein levels of p53 and p21 were unchanged and CDC20 expression increased in p53-mutant Hep3B cells with IR treatment. Over 50% of human malignancies contain a mutation in p53, including HCC 36. p53 is a key component in the DDR pathway, and mutation of P53 impairs its regulatory function in growth arrest, apoptosis, and senescence. The presence of p53 mutation in cancer is associated with a dismal prognosis 37, 38. We found that silencing of CDC20 enhanced the sensitivity to radiation in Hep3B cells mainly by amplify radiotherapy effect on growth retardation, DNA damage accumulation, apoptosis increase, and G2/M phase arrest. These findings demonstrate that CDC20 inhibition has a significant radiotherapy sensitization effect and that CDC20 might be a useful new potential therapeutic target and prognostic biomarker for HCC, especially in concert with p53 mutation.

There are some advantages of this study. First, this research contained independent HCC cohorts. Second, we demonstrated the value of significantly elevated CDC20 expression for HCC prognosis and identified CDC20 as the most valuable gene for further analysis. Third, at both the mRNA and protein levels, we validated the association between CDC20 expression and HCC prognosis. Finally, functional enrichment analysis was performed. The role of CDC20 in the infiltration of immune cells in the tumor microenvironment was demonstrated, which may guide cancer treatment and targeted drug development. However, there are also some limitations. First, we only performed analysis without evidence from cytological tests. Hence, we could not directly investigate the effect of CDC20 on the malignant behaviors of HCC. Second, the underlying mechanism of CDC20 in HCC remains unknown. In the next experiment, we will conduct further research and excavation on the specific role of CDC20 in HCC and upstream and downstream signaling pathways, and intend to select subsequent signal pathways to verify the malignant biological significance of target genes.

## Conclusion

In conclusion, we found that that CDC20 was highly expressed in HCC and participated in radio resistance of HCC cells with P53 mutation Bcl-2/Bax via signaling pathway. This study is the first to present evidence that CDC20 may play a role in improving the efficacy of radiotherapy in HCC.
